# Temporal dynamics of motion compression: a lagged extrapolation account

**DOI:** 10.1098/rsos.251638

**Published:** 2025-11-12

**Authors:** Ryohei Nakayama, Hironobu Sano, Isamu Motoyoshi

**Affiliations:** ^1^ Graduate School of Information Science and Technology, The University of Tokyo, Bunkyo-ku, Tokyo, Japan; ^2^ Department of Life Sciences, The University of Tokyo, Meguro-ku, Tokyo, Japan

**Keywords:** visual illusion, psychophysics, computational modelling

## Abstract

The visual system has been suggested to extrapolate an object’s position by integrating proximal motion signals to compensate for inevitable neural delays. This anticipatory extrapolation hypothesis is consistent with visual illusions such as the flash-lag effect, where a moving object appears ahead of a physically aligned flash, and the flash-drag effect, where the perceived position of a flash is shifted in the direction of its surrounding motion. In contrast to such motion-induced position shifts, we demonstrate an illusion in which a moving object appears to be standing still at a shifted position when surrounded by motion in the same direction. For this dissociation between perceived motion and position, we propose a computational model that incorporates the biphasic centre-surround antagonistic responses of motion detectors. In our model, positional signals derive from the temporal integration of motion-detector responses but remain unperceived during early suppression, reaching conscious perception only afterwards. The illusion was strongest when the object and surrounding motion began simultaneously, and weakened with increasing asynchrony or longer duration. The model predicts these results and accounts for several motion- and saccade-induced mislocalization phenomena, offering a unified account of dynamic position perception shaped by local and global motion signals and perceptual lag.

## 1. Introduction

**Figure 1.**
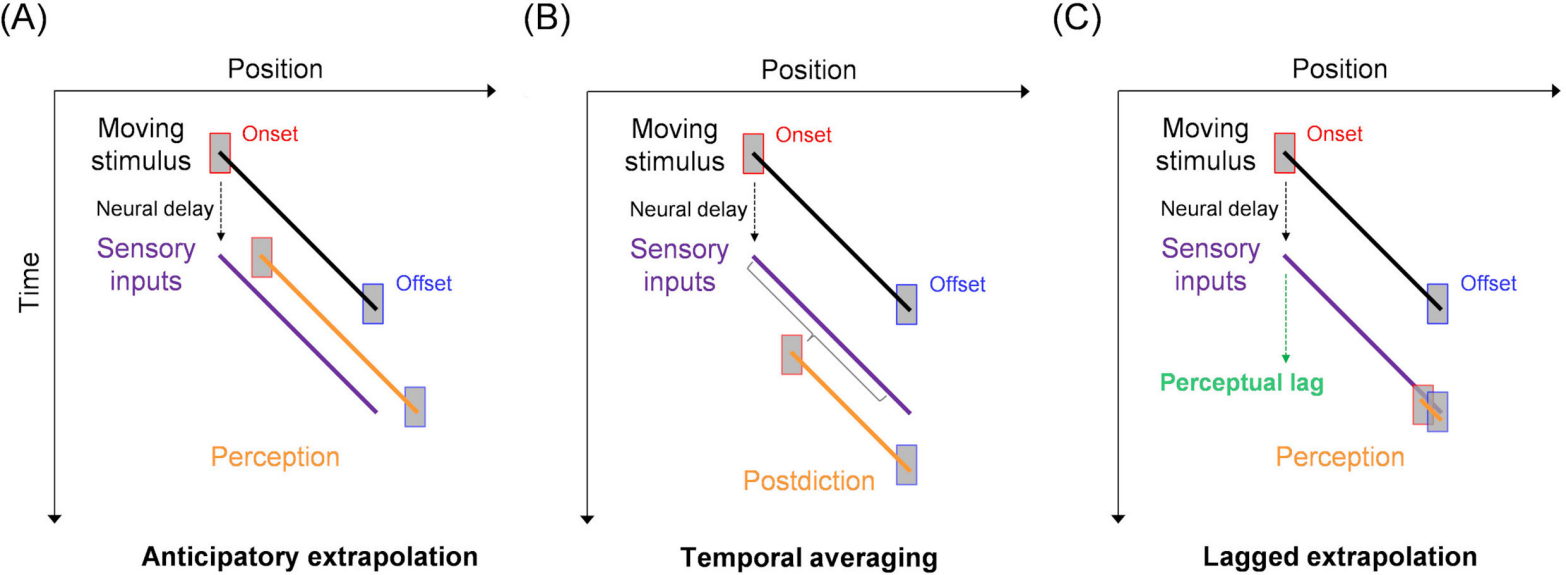
Schematic of hypotheses for position and motion perception. (A) Anticipatory extrapolation. Forward prediction to compensate for neural delays displaces perceived position in the direction of motion. (B) Temporal averaging. Post-dictive integration over a short temporal window biases perceived position towards later points along the motion trajectory. (C) Lagged extrapolation. Perceptual lag of positional signals, integrated with surrounding motion, shifts the perceived onset position in the direction of motion, while the perceived offset position remains unshifted owing to offset transients, thereby producing motion compression.

Where we perceive an object in space is strongly influenced by its dynamic environment. The visual system has been suggested to extrapolate an object’s position by integrating proximal motion signals to compensate for inevitable neural delays (figure 1A) [1–3]. This anticipatory extrapolation hypothesis is consistent with visual illusions such as the flash-lag effect, in which a moving object appears ahead of a physically aligned flash [4], and the flash-drag effect, in which the perceived position of a flash is shifted in the direction of its surrounding motion [5]. Accordingly, psychophysical studies have suggested a close link between visual motion processing and spatial localization [6].

Despite its explanatory power, the anticipatory extrapolation hypothesis has been challenged. For example, if a moving object vanishes simultaneously with the onset of a flash, the flash-lag effect is eliminated, contrary to the predictions of anticipatory extrapolation. Alternatively, it has been proposed that the perceived position of a moving object at the time of an event (such as a flash) is retrospectively attributed to its positions averaged over a brief time window following the event, a process known as temporal averaging (figure 1B) [7]. More broadly, most existing theories on motion and position perception remain limited to specific phenomena, although a comprehensive theory should account for a class of such phenomena, including mislocalization caused by saccades [8,9].

**Figure 2.**
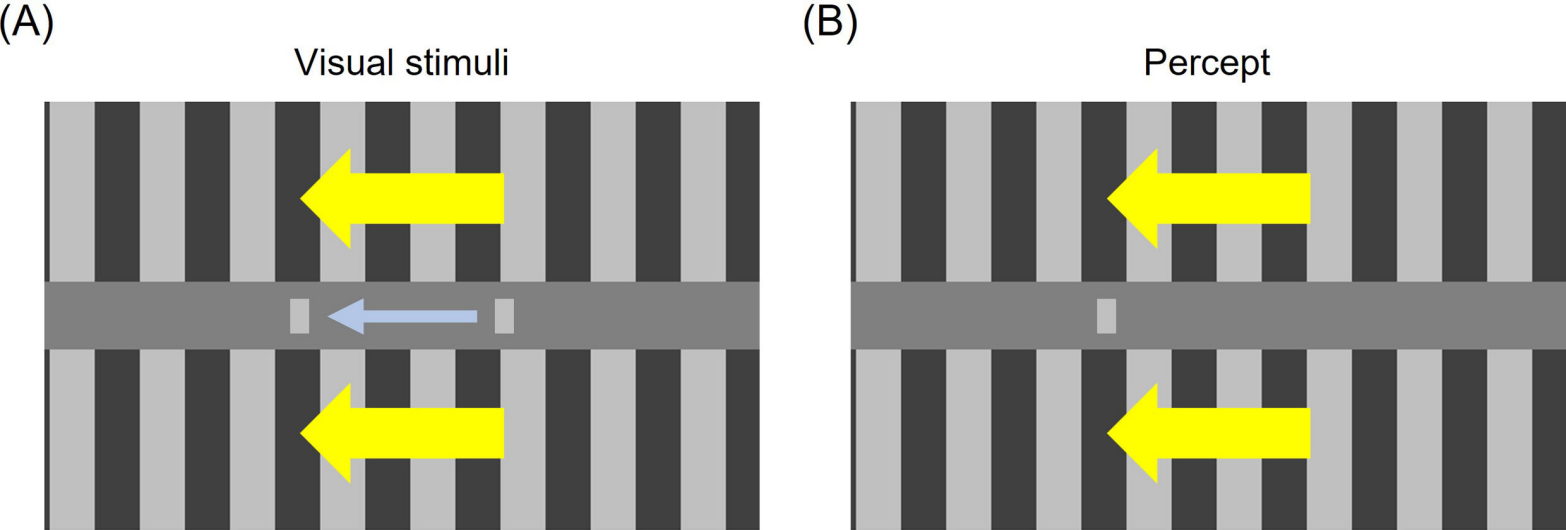
Visual stimuli and the illusion of motion compression. (A) A horizontally moving bar (target) is presented between two grating patterns (inducers) drifting in the same direction, as illustrated by the yellow and the light blue arrows, respectively. (B) The moving bar is perceived to stand still near the position where it vanishes, especially when the stimulus onset of the moving bar is synchronous with the start of the inducer motion.

To address these issues, we demonstrate a ‘motion compression’ illusion, in which a moving object appears stationary at a position shifted in the direction of its surrounding motion (figure 2; electronic supplementary material, video S1). This effect is closely related to the ‘motion freezing’ illusion, previously reported when moving objects are surrounded by global motion in the same direction [10–12], which typically involved prolonged stimulus presentations. Building on these earlier findings, we measured the perceived onset and offset positions with brief presentation durations [13], thereby systematically examining the temporal dynamics of motion compression. We found a robust compression of the onset towards the offset position that persisted for durations up to 100–130 ms.

The motion compression challenges the anticipatory extrapolation hypothesis, particularly if it predicts a greater perceived position shift along the object’s motion, integrated with the surrounding motion in the same direction. The temporal averaging hypothesis also fails to explain why the object appears completely stationary rather than moving even briefly. To the best of our knowledge, existing theories do not explicitly account for dissociations between perceived motion and position.

To address these gaps, we propose a computational model based on the biphasic centre-surround antagonistic responses of motion detectors. This ‘lagged extrapolation’ model assumes that positional signals arise from the temporal integration of motion-detector responses but remain unperceived until the suppression period ends, reaching conscious perception only afterwards (figure 1C). Despite its simplicity, as it merely posits a delay in the conscious perception of outputs from well-known motion detectors, this model successfully explains the motion compression. Furthermore, it can account for several motion- and saccade-induced mislocalization phenomena, providing a unified framework for understanding how local and global motion signals, along with perceptual lag, contribute to dynamic position perception.

## 2. Results and discussion

### 2.1. Temporal dynamics of motion compression

**Figure 3.**
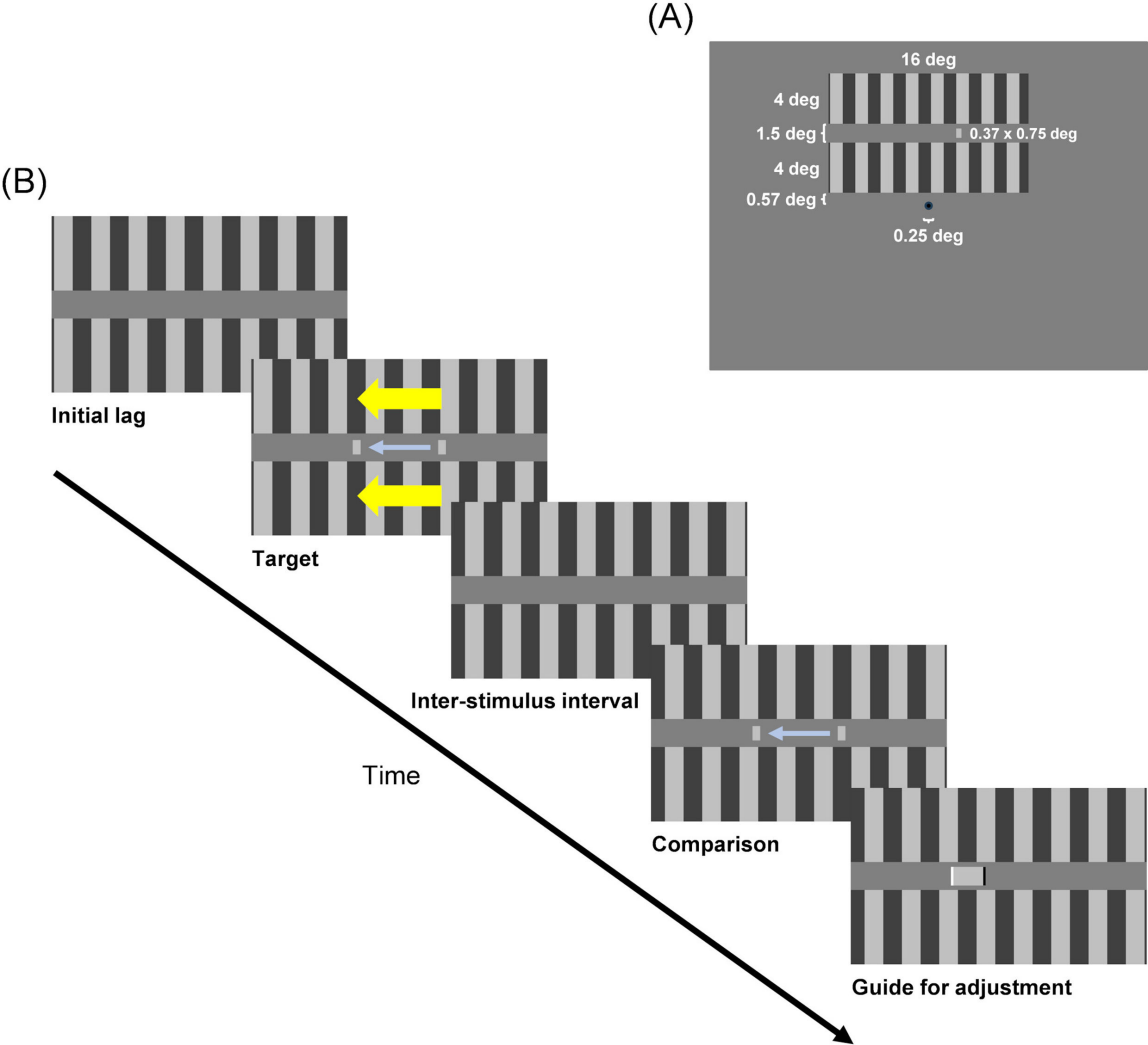
Stimulus layout (A) and sequence (B). (A) The inducers (grating patterns) and the target (grey bar) were presented above a central fixation point. (B) The display area used to present the inducers and the target is shown here. At the start of each trial, the inducers remained stationary for 333 ms (initial lag), after which they drifted at 11.2 deg s^−1^ for 1167 ms and then stopped. The target was presented between the inducers while they were drifting (or slightly before they began to drift). The target speed was 11.2 deg s^−1^ in the asynchrony and duration experiments. After a short inter-stimulus interval, the comparison was presented between the stationary inducers. Following this, the guide was presented for as long as the observers needed and was adjusted to determine the onset and offset positions of the comparison. The adjustment was performed to perceptually match the comparison motion with the target motion. The series of stimuli could be observed as many times as the observers needed.

In our experiments on motion compression, a horizontally moving target was presented between two drifting grating patterns (inducers) that moved either in the same or opposite direction relative to the target. Observers reported the perceived onset and offset positions of the target, and thus the travel distance, by adjusting the onset and offset positions of a moving probe that mimicked the target but had stationary surrounding patterns (figure 3; see §3 for details). This design ensures that any observed compression effects in the target are attributable to the presence of surrounding motion and not to generic onset mislocalization mechanisms [14], which are effectively controlled for by the probe.

**Figure 4.**
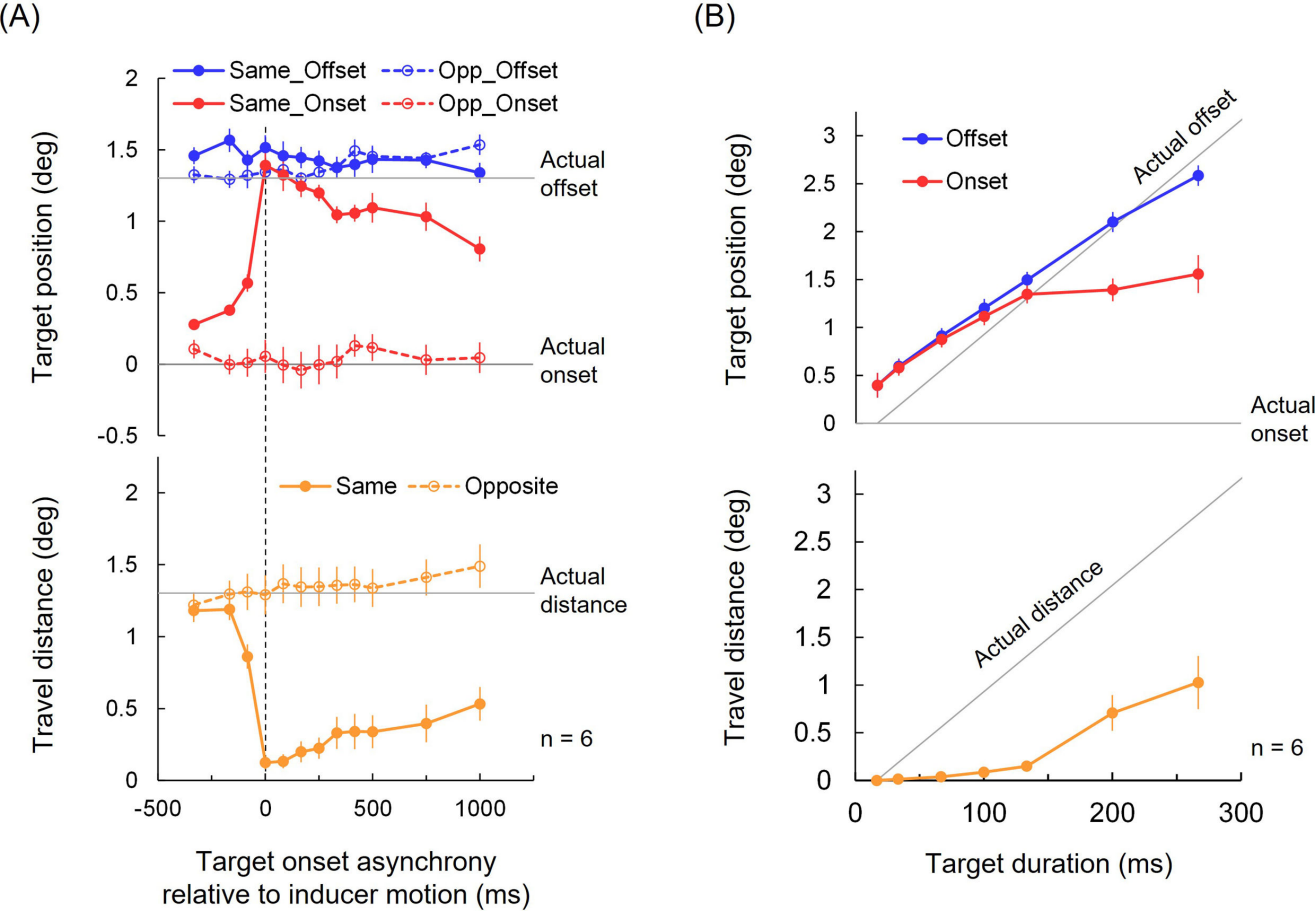
Experimental results. Red lines plot perceived onset position, blue lines plot perceived offset position, and orange lines plot perceived travel distance, as a function of target onset asynchrony from inducer motion (A) and as a function of target duration (B). Solid lines represent conditions where target and inducers moved in the same direction, while dotted lines represent conditions where they moved in opposite directions. Solid grey lines show the actual onset and offset positions as well as the actual travel distance (because the target did not move within a single frame, the actual travel distance at 17 ms is 0). Error bars represent ± 1 SE across observers.

By varying the asynchrony between the target presentation (133 ms duration) and the inducer motion (1500 ms duration), we found that in the same-direction condition, when the target presentation and inducer motion began simultaneously (0 ms asynchrony), observers perceived the onset position as shifted close to the actual offset position, while the offset position was almost correctly perceived (upper chart of figure 4A). As a result, the perceived travel distance of the target was near zero (lower chart of figure 4A). As the asynchrony increased, the motion compression diminished, and the target was perceived to travel a greater distance. Even at 0 ms asynchrony, when the target duration exceeded approximately 100–130 ms, the onset position shift reached saturation (upper chart of figure 4B), leading to the perceived travel distance increasing proportionally to the actual distance (lower chart of figure 4B), suggesting a critical duration. In the opposite-direction condition, the onset and offset positions were correctly perceived, and the motion compression was not observed (electronic supplementary material, video S2).

Consistent with these findings, in post hoc comparisons of a significant interaction between motion direction and asynchrony in perceived travel distance (lower chart of figure 4A; *F*_(11, 55)_ = 25.09, *p* < 10−17, *η*^2^ = 0.14), non-negative asynchronies (0–1000 ms) yielded significantly shorter distances than negative asynchronies (−333, −167 and −83 ms) for the same-direction condition (all *p*_Holm_ < 0.05). Within the negative range, −83 ms produced a shorter distance than −333 and −167 ms (both *p*_Holm_ < 0.05). Within the non-negative range, distances were shorter at 0–250 ms than at 1000 ms, and at 0 ms than at 750 ms (all *p*_Holm_ < 0.05). No significant difference was found for the opposite-direction condition. Post hoc comparisons of a significant duration effect (lower chart of figure 4B; *F*_(6, 30)_ = 26.61, *p* < 10−11, *η*^2^ = 0.84) showed a broadly monotonic increase: 17 ms was shorter than 67–267 ms; 33 ms was shorter than 100–267 ms; 67 ms was shorter than 133–267 ms; 100 ms was shorter than 200/267 ms; and 133 ms was shorter than 267 ms (all *p*_Holm_ < 0.05).

The motion compression exhibits strong direction selectivity, unlike visual masking, which similarly causes a loss of visibility but is non-directional [15]. This distinction underscores motion-induced suppression and positional integration as key mechanisms. The flash-drag effect depends on the asynchrony between a flash and inducer motion [16], a pattern also observed in our findings. Acceleration signals, reflected in biphasic motion-detector responses, may play a role, as reported in visual detection [17] and heading discrimination [18].

### 2.2. Lagged extrapolation as a framework for motion compression

The choice to derive positional signals from temporally integrating biphasic motion-detector responses is motivated by neurophysiological and psychophysical evidence that motion-sensitive neurons in areas such as V1 and MT exhibit biphasic temporal impulse response functions [19] and centre-surround antagonistic organization [20]. These dynamics can account for both onset–offset discrepancies and suppression effects in motion perception, which are critical for explaining the observed compression of the onset towards the offset position.

**Figure 5.**
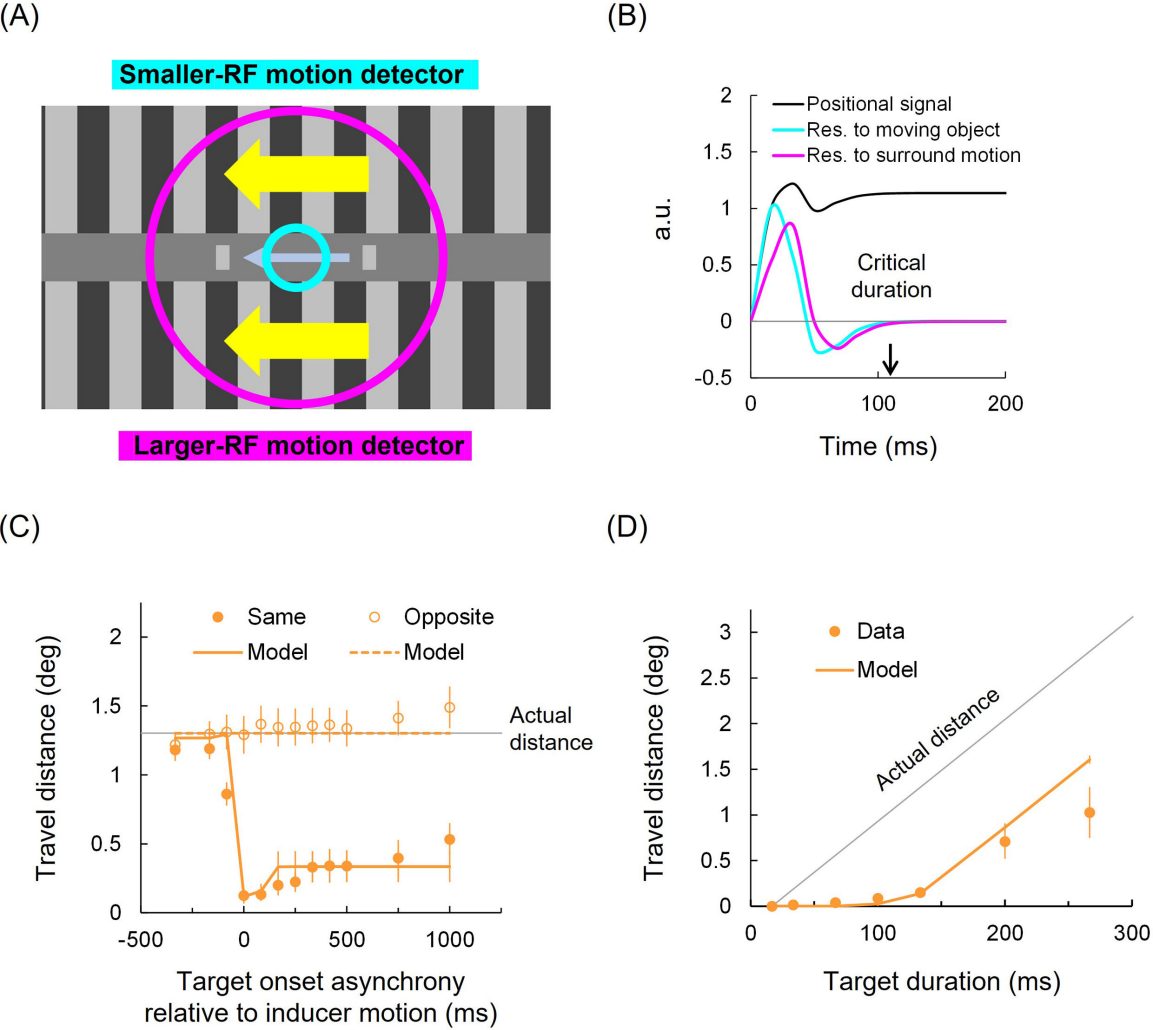
Lagged extrapolation model in motion compression. (A) Schematic of motion detectors with smaller and larger RFs tuned to a relatively small moving object and to large-field (surrounding) motion, respectively. (B) Simulated responses of the motion detectors, calculated after averaging the parameters fitted to individual data from the asynchrony experiment. (C, D) Data and model predictions in the asynchrony and duration experiments. Other conventions are the same as in figure 4.

The lagged extrapolation model assumes that positional signals are given by temporally integrating the biphasic responses of motion detectors tuned to local object motion and large-field surrounding motion, combined with the object’s initial position. As highlighted in cyan and magenta plots in figure 5A,B, each detector’s response includes a suppression period (i.e. the negative phase of biphasic responses). Additionally, the responses of local motion detectors are further suppressed by those of large-field motion detectors when both detectors receive motion inputs in the same direction, but not when the inputs are in opposite directions. The moving object is not perceived until the suppression period ends, while its positional signals continue to evolve. The object, along with its evolved positional signals, becomes consciously perceived only after the suppression period ends.

In regard to the motion compression, both the target motion and its surrounding motion contribute to shifting the neural signals of the target position in these motion directions. However, the accumulated shifts in positional signals, including those for the onset position, remain unperceived until the suppression period ends. In contrast, the offset position of a moving target is strongly signalled by the offset transients associated with the target’s abrupt vanishing, and its positional signals remain unshifted [21–23]. As a result, the perceived trajectory of the target is compressed towards its offset position, leading to the illusory perception of the moving target as stationary.

Equations (2.1) and (2.2) describe the impulse response functions of motion detectors with smaller and larger receptive fields (RFs) to the onset of a relatively small moving object and large-field motion.


(2.1) $$R_{smaller}(t)\;=\;g\left\{\frac{1}{n_{smaller}!}-\frac{B{\left(\frac{t}{T}\right)}^{2}}{\left(n_{smaller}+2\right)!}\right\}{\left(\frac{t}{T}\right)}^{n_{smaller}}e^{-\frac{t}{T}}\;$$

(2.2) $$R_{larger}\left(t\right)\;=\;g\left\{\frac{1}{n_{larger}!}-\frac{B{\left(\frac{t}{T}\right)}^{2}}{\left(n_{larger}+2\right)!}\right\}{\left(\frac{t}{T}\right)}^{n_{larger}}e^{-\frac{t}{T}}.$$

Here, *n*_smaller_, *n*_larger_, *T*, *B* and *g* are parameters that determine, respectively, the filter centre frequency for detectors with smaller and larger RFs, the tuning width in the frequency domain, the relative weight of the negative phase against the first positive phase and the gain that practically modulates the input value. This type of function has been employed in motion energy models [24,25]. The values of *n*_smaller_ and *n*_larger_ are fixed at 4 and 5 [24], while the other parameters (*T*, *B* and *g*) are free.

The response of the smaller-RF detector shifts the positional signals of a moving object, while it is suppressed by the response of the larger-RF detector [20], as described by the left subtraction term in equation (2.3). The larger-RF detector response also shifts the positional signals in proportion to the smaller-RF detector response (the right multiplication term in equation (2.3)), as surrounding motion should affect the positional signals only when it does not occur long before or after the object presentation. Temporal integration of these responses gives the positional signals, which are finally perceived when the response of the smaller-RF detector after subtracting that of the larger-RF detector asymptotically approaches zero (*k* in equation (2.4)).


(2.3) $$position=\int_{t=0}^{k}{[\left\{R_{smaller}\left(t\right)-R_{larger}\left(t+async\right)\right\}+\{R}_{larger}\left(t\right)\times R_{smaller}\left(t-async\right)\}]dt$$

(2.4) $$\;if\;t=k,R_{smaller}\left(t\right)-R_{larger}\left(t+async\right)\approx 0.$$

The model demonstrated a good fit to the results of the asynchrony experiment (average correlation, *r* = 0.93, SE = 0.02; pooled *R*^2^ = 0.73; figure 5C; see electronic supplementary material, figure S1 for individual data and electronic supplementary material, figure S2 for the data-model scatter), yielding a critical duration (i.e. *k* at *async* = 0) of 110 ms (SE = 9 ms). This aligns with the duration at which the motion compression saturates (figure 4B). On average, *T* was 0.39 (SE = 0.03), *B* was 0.72 (*SE* = 0.04) and *g* was 8.76 (SE = 0.22). Using the same model parameter values (i.e. without re-fitting), the model provided a quantitatively reasonable account for the results of the duration experiment (*r* = 0.80, SE = 0.18; pooled *R*^2^ = 0.27; figure 5D; see electronic supplementary material, figure S3 for individual data and electronic supplementary material, figure S4 for the data-model scatter). The model assumes that positional signals continue to evolve throughout the duration, but only up to the critical duration; the signals evolve until either the end of the duration or the critical duration, whichever occurs first. Since the motion direction is consistent and does not reverse, a constraint is applied: if the positional signals extend beyond the offset position, they are clamped at that position, making the perceived travel distance effectively zero.

### 2.3. Explaining various mislocalization phenomena with the lagged extrapolation

**Figure 6.**
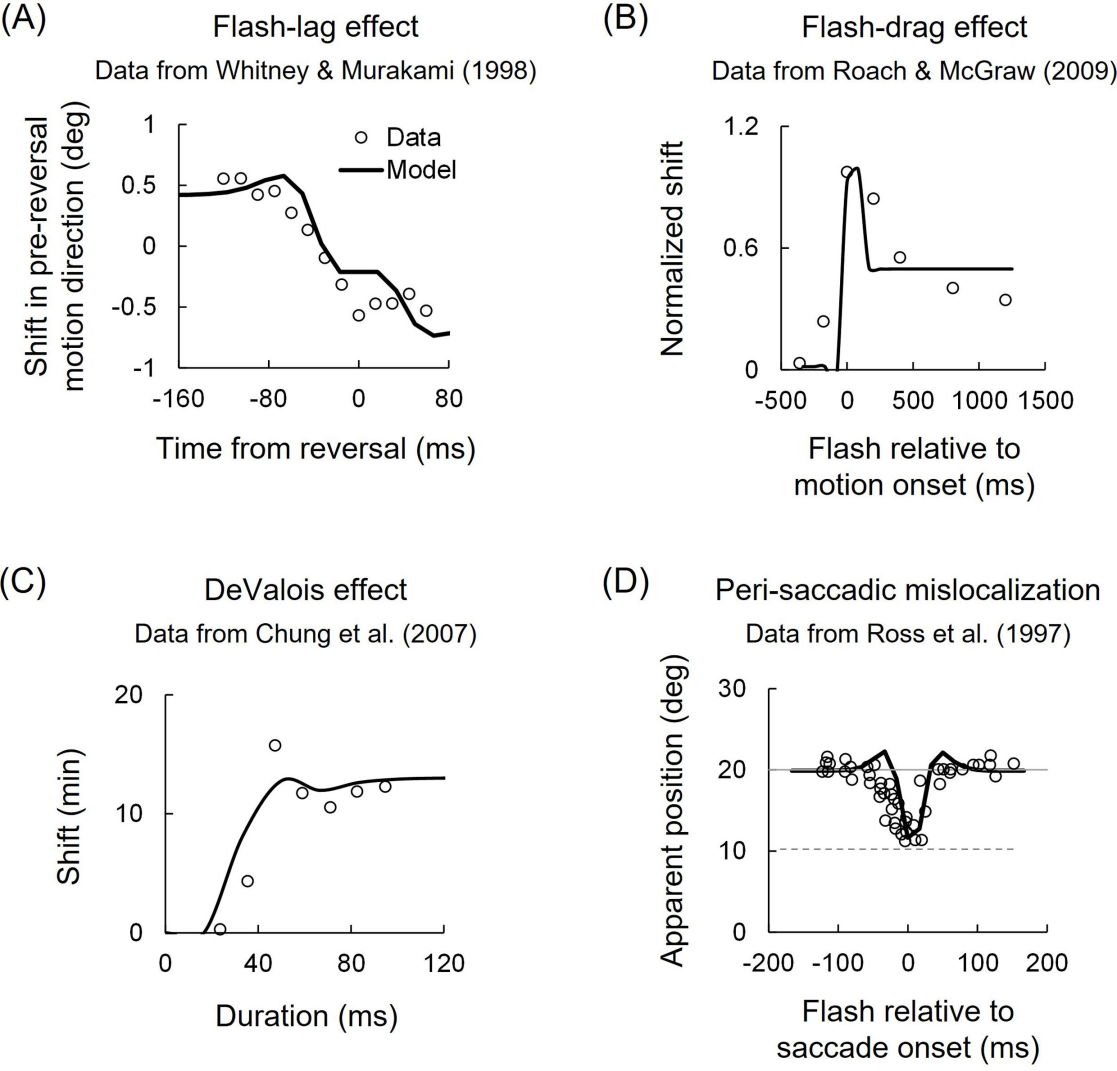
Lagged extrapolation model in various mislocalization phenomena. (A) Flash-lag effect. A perceptual position shift of a moving object in its pre-reversal motion direction relative to a flash is plotted as a function of the flash time from the motion reversal. Data from [29]. (B) Flash-drag effect. Perceptual position shift of a flash in its surrounding motion direction is plotted as a function of the flash time from the surrounding motion onset. Data from [16]. (C) DeValois effect. Perceptual position shift of a physically stationary object (envelope) in its internal pattern’s motion direction is plotted as a function of the presentation duration. Data from [30]. (D) Peri-saccadic mislocalization. The perceptual position of a flash, presented around the time of a saccade, is plotted as a function of the flash time from the saccade onset. A solid grey line indicates the actual position of the flash. A dashed grey line indicates the saccadic landing position. Data from [8].

The lagged extrapolation model accounts for perceptual position shifts reported as the flash-lag effect [4,26], flash-drag effect [5], DeValois effect [27,28] and peri-saccadic mislocalization [8,9]. Notably, the model parameters remain within the range of individual differences observed in the asynchrony experiment (electronic supplementary material, table S1), demonstrating the model’s robustness and generalizability. In the flash-lag effect, the model integrates local motion-detector responses up until the suppression period ends, causing a perceived position shift [14]. If motion reverses during this period, the reversed motion is also integrated, leading to shifts in the opposite direction at certain (nominally) pre-reversal times [29]. The effect is described by the biphasic responses of two local motion detectors tuned to opposing motion directions, with response parameters *T* = 0.52 and *B* = 0.60 (figure 6A). Similarly, the flash-drag effect occurs when a flashed object activates local motion detectors tuned to the same direction as the surrounding motion, shifting its perceived position. This effect is modelled using biphasic response parameters *T* = 0.52 and *B* = 0.87, incorporating responses from local and large-field motion detectors with different RF sizes (figure 6B). The DeValois effect, in which a stationary object appears shifted in the direction of its internal motion, follows a similar integration mechanism with parameters *T* = 0.39 and *B* = 0.72 (figure 6C). Here, internal motion is considered to play a suppressive role analogous to that of surrounding motion in the flash-drag effect, delaying object perception.

Lastly, the peri-saccadic mislocalization (under a lit environment) is modelled by integrating motion-detector responses to the rapid retinal motion caused by a saccade, leading to perceived shifts of flashed objects around the time of the saccade towards the saccadic landing position (figure 6D). This process follows the same parameter settings as the motion compression and DeValois effects (*T* = 0.39, *B* = 0.72). During the suppression period—induced by both the flash itself and retinal motion signals in the direction opposite to the saccade [31,32]—local and large-field motion-detector responses continue to integrate, resulting in a position shift perceived after suppression ends, predicting a (nominally) pre-saccadic shift. Our findings, in agreement with previous studies [33–35], suggest that retinal motion signals contribute at least partially to suppression, positional integration, and consequently peri-saccadic mislocalization, alongside extra-retinal signals.

### 2.4. Linking perceptual lag to neural dynamics and visual stability

Overall, our model suggests that dynamic position perception arises not from simple anticipatory extrapolation but rather from the temporal integration of local and global motion signals, with a lag in position and object perception. This interpretation is further supported by the results of our speed experiments, which suggest that time—rather than space—is compressed up to a critical duration (electronic supplementary material, figures S5 and S6).

The approximately 110 ms lag is broadly consistent with the neural latencies observed in motion-selective areas such as MT/V5 in macaques [36], as well as with biphasic temporal impulse response functions reported in human psychophysics both during fixation and saccades [37,38]. These parallels suggest that the model’s suppression may reflect early-to-intermediate temporal dynamics within the visual processing stream. While small-RF positional maps (e.g. V1) may encode precise onset/offset positions, intermediate/large-RF motion areas (e.g. hMT+) may integrate surrounding motion via a biphasic kernel with a time constant corresponding to the lag and feedback to bias positional readout, such that the perceived onset position is shifted in the direction of motion (whereas the offset position remains anchored). The lag also corresponds to the illusory freezing of temporal changes caused by abrupt surface completion [39], suggesting that the perceptual lag may generalize across motion and surface pattern processing.

Functionally, temporal integration during perceptual lag may serve to smooth and stabilize where we perceive an object across rapid eye movements and in complex motion scenes. The present findings enhance our understanding of the computational mechanisms underlying visual stability in more naturalistic dynamic environments than those involving a single moving object.

### 2.5. Limitations

Although the model parameters were optimized separately for each phenomenon, they consistently fell within the range of individual differences in the present experiment, suggesting shared temporal dynamics across paradigms. This consistency supports the plausibility of a common underlying mechanism rather than ad hoc curve fitting. Nevertheless, to further validate the model’s predictive power, future studies should apply parameters estimated from one paradigm to predict performance in another without re-fitting, thereby examining the limits of cross-paradigm generalization.

We instantiated two motion detectors for simplicity. In reality, position–motion integration likely reflects a population readout over diverse receptive-field sizes and spatiotemporal tuning. In this sense, our model can be viewed as a population-readout approximation reflecting a weighted combination of many responses.

We implemented the approximately 110 ms lag as fixed within individuals, yet it likely varies with multiple factors. Shorter lags, for example, under focused attention or high temporal demand, should yield weaker compression, whereas longer lags under low visibility or coherent surrounds should strengthen it, suggesting that the visual system may flexibly smooth and stabilize perceived position in dynamic environments.

Another limitation is that the model does not readily explain peri-saccadic mislocalization *opposite* to retinal motion [8]; accommodating this will likely require explicit incorporation of extra-retinal oculomotor signals and peri-saccadic remapping. Generally speaking, lagged extrapolation should operate in natural scenes beyond controlled displays. In traffic, for example, coherent surrounding flow (roadway/vehicles) would pull onset towards offset, stabilizing position under clutter but potentially underestimating early motion and biasing path-length/time-to-contact judgements. Extending the framework to cover such cases, especially under natural viewing conditions with actual eye movements, is an important direction for future work.

In our model, the position signal is not consciously accessible during the approximately 110 ms suppression period; rather, perceptual position is accessed after this delay and can be described as post-dictively updated. Importantly, this interpretation differs from temporal averaging [7], which would predict a shift towards the midpoint of the onset and offset positions rather than towards the offset itself (figure 1B). Framing the lagged extrapolation model as a variant of post-dictive position coding provides a mechanistic account of the integration dynamics and their modulation by surrounding motion. We acknowledge that the absolute timing of conscious perception was not measured directly, so the conceptual boundary is not sharp.

## 3. Methods

### 3.1. Observers

Six adults participated in all experiments (two women, four men; two authors). All observers had normal or corrected-to-normal visual acuity and provided written informed consent. All experiments were conducted in accordance with the Declaration of Helsinki (2003) and approved by the Ethics Committee for experiments on humans at the Graduate School of Arts and Sciences, University of Tokyo.

### 3.2. Apparatus

Visual stimuli were generated using MATLAB (Mathworks Inc.), Psychophysics Toolbox [40,41] and Vision Toolbox [42] programming environments. The stimuli were presented to each observer on an LCD monitor (BENQ XL2730 or BENQ XL2735) or a MacBook Pro 13-inch Retina display for an observer, in a dark room in their respective homes. The mean brightness of a uniform background ranged from 76.8 to 87.6 cd m^−2^ on the LCD monitors and was 8.5 cd m^−2^ on the MacBook Pro and was calibrated using a Colorimeter (ColorCal II CRS). The frame rate was 60 Hz, and the binocular viewing distance was set to a pixel resolution of 0.03 deg/pixel, which was 57 cm for the LCD monitors and 27 cm for the MacBook Pro.

### 3.3. Stimuli

Visual stimuli consisted of grating patterns (inducers), a target, a comparison and a guide presented in this order above a black fixation point (0.25 deg in diameter) continuously presented in the centre of the screen (figure 3).

The inducers were two vertical square-wave grating patterns (16 deg wide and 4 deg high; 0.5 Michelson luminance contrast) with a vertical gap of 1.5 deg from each other. The spatial frequency was 0.5 cycles per degree, with an initial phase randomly determined at the start of each experimental session. The lower edge of the lower inducer was 0.57 deg above the fixation point. The inducers were stationary for the first 333 ms, after which they drifted for 1167 ms (until the end of the first 1500 ms). After stopping, inducers remained stationary, and at the start of the next observation (or the next trial), the inducers began to drift from the phase at which the inducers had stopped in the last observation. To avoid the aftereffect of motion adaptation, the drifting direction of the inducers was switched between leftward and rightward from trial to trial.

The target was a stationary or moving bar (0.37 deg wide and 0.75 deg high; 0.5 luminance contrast) presented at half the distance between the inducers while they were drifting (or slightly before they began to drift). Target onset asynchrony relative to the start of the inducer motion, target duration, and target speed are experimental conditions (see electronic supplementary material, figures S5 and S6 for the results of the speed experiments).

The comparison was identical to the target (including the presentation duration) except for the onset and offset positions (and thus speed), which were determined by adjusting the guide. In the first observation of each trial, when no adjustment had yet been made, a stationary bar was presented above the fixation point.

The guide was a light grey rectangle (1.12 deg high; 0.3 luminance contrast) presented at half the distance between the stationary inducers. One edge of the rectangle was black, and the other was white. The black edge corresponded to the onset position, the white edge to the offset position (and thus the grey part to the travel distance) of the comparison. In the first observation of each trial, when no adjustment had yet been made, a light grey line (1 pixel wide and 1.12 deg high) was presented above the fixation point.

### 3.4. Procedure

Because the illusion was expected to occur robustly, the experiment was conducted using an adjustment method. Observers were instructed to view the stimuli while maintaining fixation on the fixation point and to adjust the position and travel distance of the guide so that the target and comparison stimuli perceptually matched in motion. By pressing the corresponding buttons, observers could adjust the guide (and thus the comparison) by 0.06 deg at a time, either to the left or right for the position or by elongating or shortening for travel distance. Observers were able to view the series of stimuli as many times as necessary, and the adjustment of the guide was reflected in the comparison in the next presentation. Observers finally decided the match between the target and comparison stimuli, and the next trial began soon after.

Each session consisted of 35–60 trials and was repeated so that 10 trials per condition were collected for each observer. All observers had one or a few practice sessions of seven trials prior to the experimental sessions. Observers were allowed to use the guide when adjusting the comparison position and travel distance, but were instructed to make their final decision based on the observations of the target and comparison stimuli without referencing the guide.

In the asynchrony experiment, the same and opposite motion directions of the target relative to the drifting direction of the inducers were conditioned in separate sessions (i.e. the same and opposite directions were not mixed in one session). The asynchrony was either −333, −167, −83, 0, 83, 167, 250, 333, 417, 500, 750 or 1000 ms. In the negative asynchrony conditions, the target was onset before the inducers began to drift. These asynchrony conditions were randomly interleaved within a session. The target duration was 133 ms, and the target speed was identical to that of the inducers (11.2 deg s^−1^) regardless of the motion direction.

In the duration experiment, the target duration was either 17, 33, 67, 100, 133, 200 or 267 ms. These duration conditions were randomly interleaved within a session. The target onset was synchronized to the start of the inducer motion (0 ms asynchrony), as this produced the most pronounced effect in the asynchrony experiment. The target speed was matched to the inducer speed (11.2 deg s^−1^). The travel distance of the target was changed in proportion to the target duration to 0, 0.19, 0.56, 0.93, 1.30, 2.05 or 2.79 deg. In the 17-ms (one screen frame) condition, the target was a stationary flash. The target and the inducers moved in the same direction for all measurements in the duration experiment.

### 3.5. Analysis

For group-level statistical analyses of the asynchrony experiment data, two-way repeated-measures analysis of variance (ANOVA) was performed for the travel distance values with motion direction and asynchrony as factors. All the multiple comparisons between asynchronies combined with motion directions were corrected using Holm’s method. For the duration experiment, one-way repeated-measures ANOVA was performed for the travel distance values with duration as a factor. All the multiple comparisons between durations were corrected using Holm’s method. The family-wise significance level (*α*) was set at 0.05.

The lagged extrapolation model was fit to the asynchrony experiment data using the least-squares method. Model accuracy was summarized by the pooled coefficient of determination (*R*^²^), calculated as $1-\frac{\sum SS_{\text{res}}}{\sum SS_{\text{tot}}}$, where $SS_{\text{res}}=\sum (y-\hat{y}{)}^{2}$and $SS_{\text{tot}}=\sum (y-\overset{ˉ}{y}{)}^{2}$. The same parameter values were then applied to the duration experiment data, and parameter values within the range of individual differences observed in the asynchrony experiment were applied to datasets from previous studies.

## Data Availability

All data supporting the findings of this study and the analysis code are publicly available via the following figshare link: https://figshare.com/s/cb61fbdf7a105af36711. Supplementary material is available online [43].
